# Supplementary material for: Challenging Cases of Aortic Prosthesis Dysfunction, the Importance of Multimodality Imaging, a Case Series
Source: Diagnostics (Basel). 2021 Dec 8;11(12):2305. doi: 10.3390/diagnostics11122305 (PMC8700716; doi:10.3390/diagnostics11122305)
Supplement: Supplementary file 1 [file diagnostics-11-02305-s001.zip › diagnostics-1427967-supplementary.pdf]

Supplementary Materials

Supplementary Table S1. Body surface area (BSA-m^2), tube voltage (TV-kW) and dose–length product (DLP-mGy*cm) in each patient.

	BSA	TV	DLP
Case 1	1.45	80	304.1
Case 2	1.58	80	113.7
Case 3	2.04	120	1056
Case 4	1.88	100	729.3
